# Supplementary material for: A revised model for promoter competition based on multi-way chromatin interactions at the α-globin locus
Source: Nat Commun. 2019 Nov 27;10:5412. doi: 10.1038/s41467-019-13404-x (PMC6881440; doi:10.1038/s41467-019-13404-x)
Supplement: Supplementary file 1 — Supplementary Information [file 41467_2019_13404_MOESM1_ESM.pdf]

SUPPLEMENTARY INFORMATION FOR:

A revised model for promoter competition based on multi-way chromatin interactions at the α-globin locus

Oudelaar *et al.*

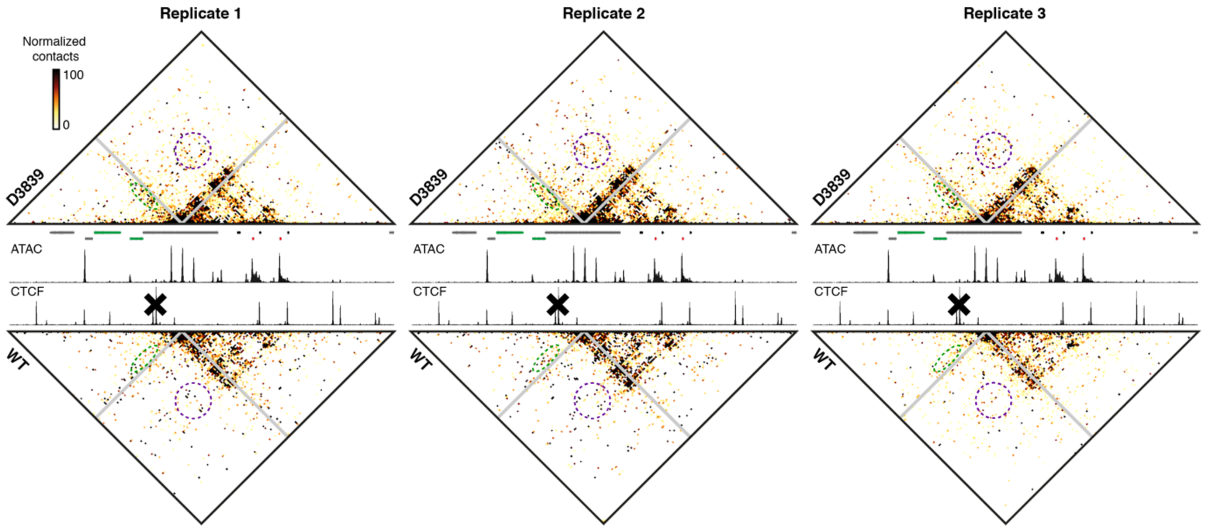

Supplementary Figure 1: Reproducibility of R2 Tri-C contact matrices. Tri-C contact matrices showing multi-way chromatin interactions with R2 in individual biological replicates of D3839 (top) and WT (bottom) erythroid cells. Matrices represent normalized, unique contact counts at 1 kb resolution with proximity contacts around the R2 viewpoint excluded (gray diagonal). Individual replicates show similar patterns of increased R2 interactions with R1 and the *Mpg/Rhbdf1* promoters (green) and with the *Mpg/Rhbdf1* promoters and the α-globin promoters (purple) in the D3839 cells compared to WT cells. Stratum-adjusted correlation coefficients as determined by HiCRep are 0.94-0.97 for D3839 and 0.85-0.92 for WT matrices. Gene annotation, open chromatin (ATAC) and CTCF occupancy in WT erythroid cells are shown in the middle. Coordinates (mm9): chr11:32,070,000–32,250,000.

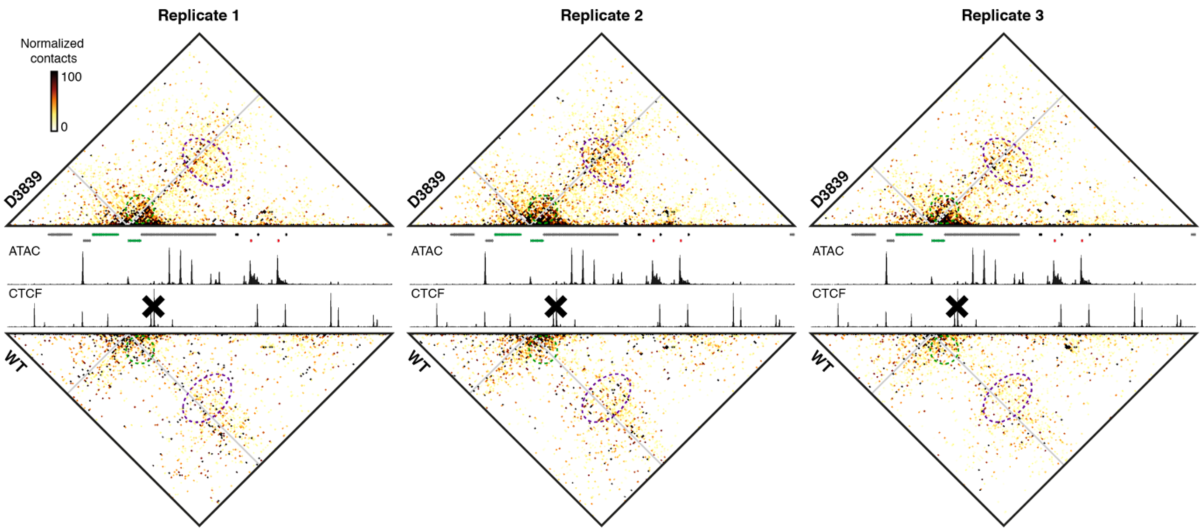

Supplementary Figure 2: Reproducibility of *Mpg* Tri-C contact matrices. Tri-C contact matrices showing multi-way chromatin interactions with *Mpg* in individual biological replicates of D3839 (top) and WT (bottom) erythroid cells. Matrices represent normalized, unique contact counts at 1 kb resolution with proximity contacts around the *Mpg* viewpoint excluded (gray diagonal). Individual replicates show similar patterns of increased proximal *Mpg* interactions, including with R1 and the *Rhbdf1* promoter (green) and with the *Rhbdf1* promoter and the α-globin enhancers/promoters (purple) in the D3839 cells compared to WT cells. Stratum-adjusted correlation coefficients as determined by HiCRep are 0.98-0.99 for D3839 and 0.93-0.95 for WT matrices. Gene annotation, open chromatin (ATAC) and CTCF occupancy in WT erythroid cells are shown in the middle. Coordinates (mm9): chr11:32,070,000–32,250,000.

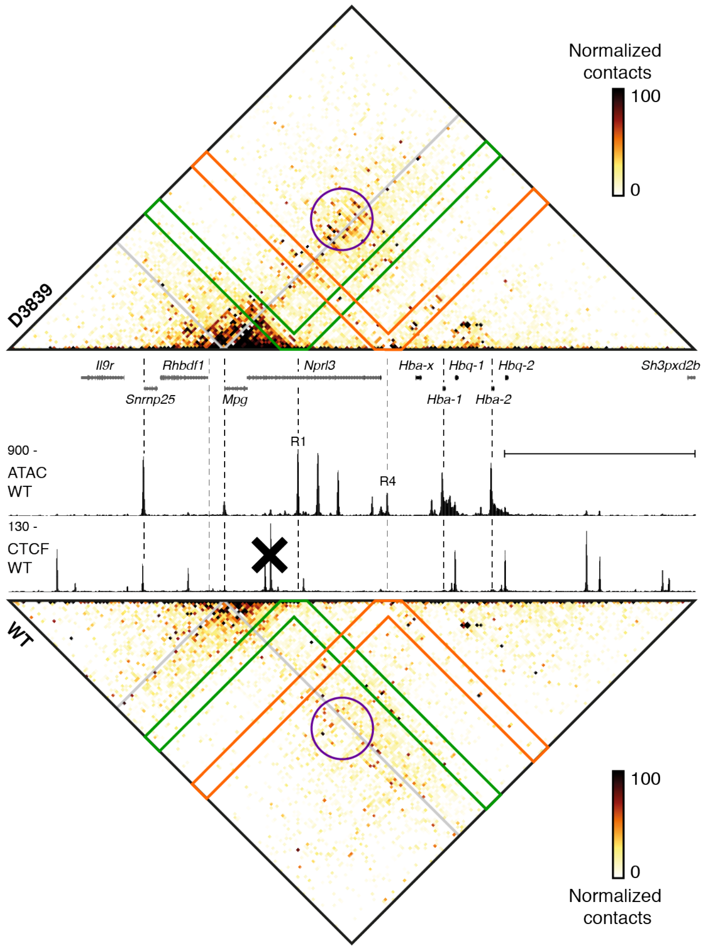

Supplementary Figure 3: Multi-way interactions with the *Mpg* promoter. Tri-C contact matrices showing multi-way chromatin interactions with *Mpg* in D3839 (top) and WT (bottom) erythroid cells. Matrices represent mean numbers of normalized, unique contact counts at 1 kb resolution in n=3 biological replicates with proximity contacts around the *Mpg* viewpoint excluded (gray diagonal). Gene annotation, open chromatin (ATAC) and CTCF occupancy in WT erythroid cells are shown in the middle. Coordinates (mm9): chr11:32,070,000–32,250,000. To emphasize that the *Mpg* promoter preferentially interacts with the α-globin enhancers in a complex which includes the α-globin and *Rhbdf1* promoters, we have highlighted the regions of the contact matrices that show all the multi-way interactions between *Mpg* and R1 (green) and between *Mpg* and R4 (orange). When *Mpg* interacts with R1 or R4 in D3839 cells, there are clear enrichments over the other α-globin enhancers and the α-globin and *Rhbdf1* promoters, indicating that *Mpg* preferentially interacts with these elements in a complex. The formation of a structure in which multiple promoters interact together is also evident from the increased *Mpg* interactions with the α-globin and *Rhbdf1* promoters (purple) in D3839 cells.

	NlaIII fragment coordinates	NlaIII fragment size (bp)
**R2**	chr11:32,150,926-32,151,102	176
**Mpg**	chr11:32,126,462-32,126,692	230

Supplementary Table 1. Tri-C viewpoints. Overview of the coordinates and sizes of the restriction fragments used as viewpoints in the Tri-C experiments. The oligonucleotide pools we used for viewpoint enrichment also contained oligonucleotides targeting the following NlaIII fragments: chr11:32137188-32137324, chr11:32100062-32100217 and chr11:32160146-32160318. These restriction fragments were excluded from analysis to prevent artefacts.

	Sequence
**R2**	GGTCAAAGTAGCATACACCCATCTGGAACCTATCAGTGACCATAGTCAACAGCAGGTGTACACA CCCAGGCCAAGGGTGGAGCAGACCACTGTGGGATCTATGGAGATGCTTGAACGAGC
**Mpg**	TCCGGTGGCCTGGCCTGTGCTGGCGGCGACTAGATGCCCGCGCGGTGGTAGTGCGCGCCC GGGCAGAGGAGCCCTAAAACCGGTGTCCGTGACCCTGCTCCCCGACACCGAGCAGCCT

Supplementary Table 2. Tri-C capture oligonucleotides. Overview of the sequences of the Tri-C capture oligonucleotides used to enrich for viewpoints of interest. These 120 bp sequences were designed to target the middle of the restriction fragments listed in Supplementary Table 1.
